# 3-Methyl-5-oxo-4-(2-phenyl­hydrazinyl­idene)-4,5-dihydro-1*H*-pyrazole-1-carbothio­amide

**DOI:** 10.1107/S1600536811009779

**Published:** 2011-03-19

**Authors:** Hoong-Kun Fun, Safra Izuani Jama Asik, Ibrahim Abdul Razak, Shobhitha Shetty, Balakrishna Kalluraya

**Affiliations:** a X-ray Crystallography Unit, School of Physics, Universiti Sains Malaysia, 11800 USM, Penang, Malaysia; b Department of Studies in Chemistry, Mangalore University, Mangalagangotri, Mangalore 574 199, India

## Abstract

In the title compound, C_11_H_11_N_5_OS, the pyrazole ring is approximately planar, with a maximum deviation of 0.010 (2) Å. The dihedral angles between the benzene ring and the pyrazole and carbothio­amide groups are 5.42 (9) and 10.61 (18)°, respectively. An intra­molecular N—H⋯O hydrogen bond generates an *S*(6) ring motif. In the crystal, mol­ecules are connected by inter­molecular N—H⋯O and C—H⋯S hydrogen bonds, forming *R*_2_^2^(12) ring motifs. In addition, there is a π–π stacking inter­action [centroid–centroid distance = 3.5188 (11) Å] between the pyrazole and benzene rings. These inter­actions link the mol­ecules into infinite chains along [001].

## Related literature

For general background to and applications of pyrazole derivatives, see: Rai & Kalluraya (2006 ▶); Rai *et al.* (2008 ▶); Sridhar & Perumal (2003 ▶). For graph-set theory, see: Bernstein *et al.* (1995 ▶). For bond-length data, see: Allen *et al.* (1987 ▶).
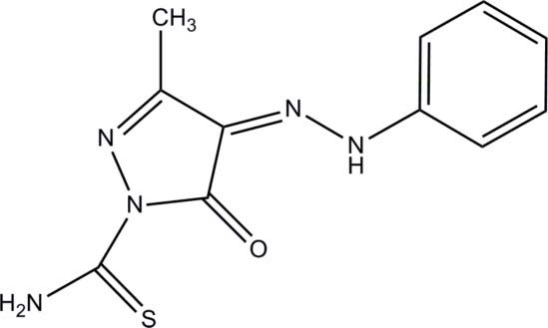


## Experimental

### 

#### Crystal data

C_11_H_11_N_5_OS
*M* *_r_* = 261.31
Monoclinic, 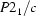

*a* = 7.7388 (1) Å
*b* = 16.1103 (3) Å
*c* = 11.3575 (2) Å
β = 121.058 (1)°
*V* = 1213.00 (3) Å^3^
*Z* = 4
Mo *K*α radiation
μ = 0.26 mm^−1^
*T* = 296 K
0.53 × 0.39 × 0.13 mm

#### Data collection

Bruker SMART APEXII CCD area-detector diffractometer
Absorption correction: multi-scan (*SADABS*; Bruker, 2009 ▶) *T*_min_ = 0.874, *T*_max_ = 0.967
16258 measured reflections
4393 independent reflections
3037 reflections with *I* > 2σ(*I*)
*R*_int_ = 0.026

#### Refinement

*R*[*F* ^2^ > 2σ(*F* ^2^)] = 0.049
*wR*(*F* ^2^) = 0.135
*S* = 1.05
4393 reflections
164 parameters
H-atom parameters constrained
Δρ_max_ = 0.33 e Å^−3^
Δρ_min_ = −0.39 e Å^−3^

### 

Data collection: *APEX2* (Bruker, 2009 ▶); cell refinement: *SAINT* (Bruker, 2009 ▶); data reduction: *SAINT*; program(s) used to solve structure: *SHELXTL* (Sheldrick, 2008 ▶); program(s) used to refine structure: *SHELXTL*; molecular graphics: *SHELXTL*; software used to prepare material for publication: *SHELXTL* and *PLATON* (Spek, 2009 ▶).

## Supplementary Material

Crystal structure: contains datablocks global, I. DOI: 10.1107/S1600536811009779/sj5118sup1.cif

Structure factors: contains datablocks I. DOI: 10.1107/S1600536811009779/sj5118Isup2.hkl

Additional supplementary materials:  crystallographic information; 3D view; checkCIF report

## Figures and Tables

**Table 1:** Hydrogen-bond geometry (Å, °)

*D*—H⋯*A*	*D*—H	H⋯*A*	*D*⋯*A*	*D*—H⋯*A*
N1—H1*B*⋯O1	0.86	2.16	2.8147 (16)	132
N5—H5*C*⋯O1^i^	0.86	2.03	2.8806 (15)	172
C1—H1*A*⋯S1^ii^	0.93	2.80	3.6838 (16)	159

Symmetry codes: (i) 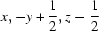; (ii) 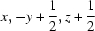.

**Fig. 1.**
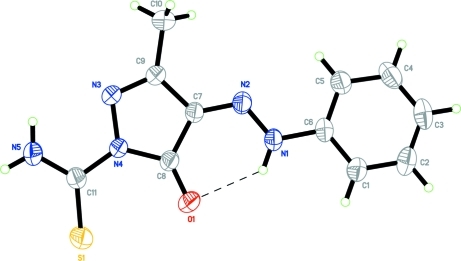
The structure of (I), showing 50% probability displacement ellipsoids and the atom-numbering scheme.

**Fig. 2.**
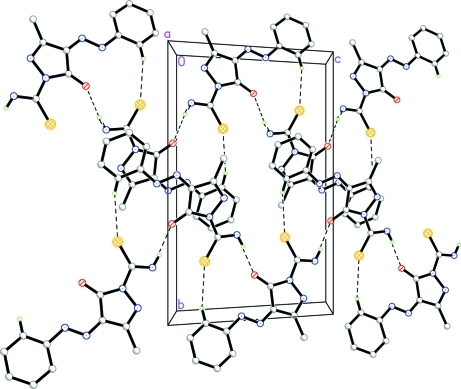
The crystal packing of (I) viewed along the a axis, showing infinite one-dimensional chains along [0 0 1]. Hydrogen bonds are shown as dashed lines.

## supplementary crystallographic information

### Comment

Pyrazole derivatives are in general well-known nitrogen-containing heterocyclic compounds and various procedures have been developed for their synthesis (Rai & Kalluraya, 2006). The chemistry of pyrazole derivatives has been the subject of much interest due to their importance for various applications and their widespread potential and proven biological and pharmacological activities (Rai *et al.*, 2008). Steroids containing a pyrazole moiety are of interest as psychopharmacological agents. Some alkyl- and aryl-substituted pyrazoles have a sharply pronounced sedative action on the central nervous system. Furthermore, certain alkyl pyrazoles show significant bacteriostatic, bacteriocidal, fungicidal, analgesic and anti-pyretic activities (Sridhar & Perumal, 2003).

Fig.1 shows the molecular structure of (I). The pyrazole (C7–C9/N3/N4) ring is approximately planar with a maximum deviation of 0.010 (2) Å for atom C8. The dihedral angle between benzene and pyrazole rings is 5.42 (9)°. The carbothioamide group (S1/C11/N5) is twisted at a dihedral angle 10.61 (18)° from the pyrazole ring. The bond lengths (Allen *et al.*, 1987) in (I) show normal values. An intramolecular N1—H1B···O1 hydrogen bond (Table 1) generates an S(6) ring motif (Bernstein *et al.*, 1995).

In the crystal packing of (I) (Fig. 2), molecules are connected by N5—H5C···O1 and C1—H1A···S1 intermolecular hydrogen bonds to form R^2^_2_(12) ring motifs. These interactions also link the molecules into infinite one-dimensional chains along [0 0 1]. In addition, there is a π–π stacking interaction between pyrazole (C7–C9/N3/N4; centroid *Cg*1) and benzene (C1–C6; centroid *Cg*2) rings with a *Cg*1···*Cg*2 separation of  3.5188 (11) Å.

### Experimental

To a solution of ethyl-3-oxo-2-(2-phenylhydrazinylidene) butanoate (0.01mol) dissolved in glacial acetic acid (20ml), a solution of thiosemicarbazide (0.02mol) in glacial acetic acid (25ml) was added and the mixture was refluxed for 4 h. It is cooled and allowed to stand overnight. The solid product that separated out was filtered and dried. It was then recrystallized from ethanol. Crystals suitable for X-ray analysis were obtained from 1:2 mixtures of DMF and ethanol by slow evaporation.

### Refinement

All the H atoms were placed in calculated positions with N—H = 0.86Å, C—H = 0.93Å, and for C—H_3_ = 0.96Å. The *U*_iso_ values were constrained to be 1.5*U*_eq_ of the carrier atom for methyl H atoms and 1.2*U*_eq_ for the remaining H atoms. A rotating group model was used for the methyl groups.

### Crystal data

**Table:**

C_11_H_11_N_5_OS	*F*(000) = 544
*M**_r_* = 261.31	*D*_x_ = 1.431 Mg m^−^^3^
Monoclinic, *P*2_1_/*c*	Mo *K*α radiation, λ = 0.71073 Å
Hall symbol: -P 2ybc	Cell parameters from 5770 reflections
*a* = 7.7388 (1) Å	θ = 2.5–31.9°
*b* = 16.1103 (3) Å	µ = 0.26 mm^−^^1^
*c* = 11.3575 (2) Å	*T* = 296 K
β = 121.058 (1)°	Plate, orange
*V* = 1213.00 (3) Å^3^	0.53 × 0.39 × 0.13 mm
*Z* = 4	

### Data collection

**Table:**

Bruker SMART APEXII CCD area-detector diffractometer	4393 independent reflections
Radiation source: sealed tube	3037 reflections with *I* > 2σ(*I*)
graphite	*R*_int_ = 0.026
φ and ω scans	θ_max_ = 32.6°, θ_min_ = 2.5°
Absorption correction: multi-scan (*SADABS*; Bruker, 2009)	*h* = −11→11
*T*_min_ = 0.874, *T*_max_ = 0.967	*k* = −24→23
16258 measured reflections	*l* = −17→16

### Refinement

**Table:**

Refinement on *F*^2^	Primary atom site location: structure-invariant direct methods
Least-squares matrix: full	Secondary atom site location: difference Fourier map
*R*[*F*^2^ > 2σ(*F*^2^)] = 0.049	Hydrogen site location: inferred from neighbouring sites
*wR*(*F*^2^) = 0.135	H-atom parameters constrained
*S* = 1.05	*w* = 1/[σ^2^(*F*_o_^2^) + (0.0587*P*)^2^ + 0.2537*P*] where *P* = (*F*_o_^2^ + 2*F*_c_^2^)/3
4393 reflections	(Δ/σ)_max_ < 0.001
164 parameters	Δρ_max_ = 0.33 e Å^−^^3^
0 restraints	Δρ_min_ = −0.39 e Å^−^^3^

### Special details

**Table:**

Geometry. All esds (except the esd in the dihedral angle between two l.s. planes) are estimated using the full covariance matrix. The cell esds are taken into account individually in the estimation of esds in distances, angles and torsion angles; correlations between esds in cell parameters are only used when they are defined by crystal symmetry. An approximate (isotropic) treatment of cell esds is used for estimating esds involving l.s. planes.
Refinement. Refinement of F^2^ against ALL reflections. The weighted R-factor wR and goodness of fit S are based on F^2^, conventional R-factors R are based on F, with F set to zero for negative F^2^. The threshold expression of F^2^ > 2sigma(F^2^) is used only for calculating R-factors(gt) etc. and is not relevant to the choice of reflections for refinement. R-factors based on F^2^ are statistically about twice as large as those based on F, and R- factors based on ALL data will be even larger.

### Fractional atomic coordinates and isotropic or equivalent isotropic displacement parameters (Å^2^)

**Table:**

	*x*	*y*	*z*	*U*_iso_*/*U*_eq_	
S1	0.35472 (10)	0.29034 (2)	0.30227 (5)	0.07026 (19)	
O1	0.36818 (17)	0.14963 (6)	0.49606 (10)	0.0434 (2)	
N1	0.26541 (17)	0.00165 (7)	0.57723 (11)	0.0346 (2)	
H1B	0.3036	0.0523	0.5989	0.041*	
N2	0.23279 (17)	−0.02868 (7)	0.46071 (11)	0.0340 (2)	
N3	0.25183 (18)	0.05536 (7)	0.18342 (11)	0.0340 (2)	
N4	0.30783 (17)	0.12552 (6)	0.27173 (11)	0.0324 (2)	
N5	0.3303 (2)	0.19488 (8)	0.10601 (13)	0.0478 (3)	
H5B	0.3161	0.1470	0.0685	0.057*	
H5C	0.3439	0.2384	0.0678	0.057*	
C1	0.2876 (2)	−0.01706 (10)	0.79423 (14)	0.0413 (3)	
H1A	0.3376	0.0367	0.8182	0.050*	
C2	0.2625 (3)	−0.06542 (11)	0.88499 (15)	0.0494 (4)	
H2A	0.2961	−0.0441	0.9703	0.059*	
C3	0.1883 (3)	−0.14496 (11)	0.84978 (17)	0.0509 (4)	
H3A	0.1732	−0.1775	0.9115	0.061*	
C4	0.1363 (3)	−0.17618 (10)	0.72246 (19)	0.0523 (4)	
H4A	0.0839	−0.2296	0.6983	0.063*	
C5	0.1611 (2)	−0.12903 (9)	0.62989 (16)	0.0433 (3)	
H5A	0.1272	−0.1505	0.5446	0.052*	
C6	0.23757 (19)	−0.04923 (8)	0.66727 (13)	0.0337 (3)	
C7	0.25637 (19)	0.01943 (7)	0.37735 (12)	0.0308 (2)	
C8	0.31689 (19)	0.10634 (8)	0.39492 (12)	0.0313 (3)	
C9	0.2218 (2)	−0.00556 (8)	0.24546 (13)	0.0328 (3)	
C10	0.1592 (3)	−0.08981 (9)	0.18412 (16)	0.0481 (4)	
H10A	0.0995	−0.0862	0.0863	0.072*	
H10B	0.0625	−0.1121	0.2046	0.072*	
H10C	0.2751	−0.1255	0.2219	0.072*	
C11	0.3313 (2)	0.20147 (8)	0.22208 (14)	0.0356 (3)	

### Atomic displacement parameters (Å^2^)

**Table:**

	*U*^11^	*U*^22^	*U*^33^	*U*^12^	*U*^13^	*U*^23^
S1	0.1363 (5)	0.0316 (2)	0.0759 (3)	−0.0167 (2)	0.0783 (4)	−0.01097 (18)
O1	0.0638 (7)	0.0371 (5)	0.0345 (5)	−0.0008 (4)	0.0291 (5)	−0.0053 (4)
N1	0.0439 (6)	0.0335 (5)	0.0316 (5)	0.0008 (4)	0.0233 (5)	0.0044 (4)
N2	0.0390 (6)	0.0347 (5)	0.0320 (5)	0.0033 (4)	0.0210 (5)	0.0043 (4)
N3	0.0454 (6)	0.0316 (5)	0.0304 (5)	−0.0027 (4)	0.0233 (5)	−0.0037 (4)
N4	0.0459 (6)	0.0276 (5)	0.0302 (5)	−0.0019 (4)	0.0242 (5)	−0.0015 (4)
N5	0.0785 (10)	0.0355 (6)	0.0431 (6)	−0.0039 (6)	0.0411 (7)	0.0037 (5)
C1	0.0478 (8)	0.0442 (7)	0.0362 (6)	−0.0027 (6)	0.0247 (6)	0.0024 (6)
C2	0.0543 (9)	0.0645 (10)	0.0351 (7)	0.0010 (7)	0.0271 (7)	0.0084 (7)
C3	0.0547 (9)	0.0569 (9)	0.0507 (8)	0.0075 (7)	0.0339 (7)	0.0219 (7)
C4	0.0645 (10)	0.0377 (7)	0.0662 (10)	0.0024 (7)	0.0420 (9)	0.0121 (7)
C5	0.0561 (9)	0.0370 (7)	0.0448 (8)	0.0019 (6)	0.0318 (7)	0.0042 (6)
C6	0.0345 (6)	0.0375 (6)	0.0330 (6)	0.0050 (5)	0.0202 (5)	0.0091 (5)
C7	0.0368 (6)	0.0298 (5)	0.0298 (5)	0.0014 (5)	0.0201 (5)	0.0018 (4)
C8	0.0381 (6)	0.0309 (5)	0.0297 (5)	0.0024 (5)	0.0208 (5)	0.0004 (4)
C9	0.0397 (7)	0.0310 (6)	0.0314 (6)	−0.0003 (5)	0.0210 (5)	−0.0015 (4)
C10	0.0700 (10)	0.0342 (7)	0.0471 (8)	−0.0090 (6)	0.0351 (8)	−0.0083 (6)
C11	0.0441 (7)	0.0307 (6)	0.0377 (6)	−0.0003 (5)	0.0252 (6)	0.0024 (5)

### Geometric parameters (Å, °)

**Table:**

S1—C11	1.6560 (13)	C1—H1A	0.9300
O1—C8	1.2218 (15)	C2—C3	1.377 (3)
N1—N2	1.3073 (15)	C2—H2A	0.9300
N1—C6	1.4111 (16)	C3—C4	1.380 (3)
N1—H1B	0.8600	C3—H3A	0.9300
N2—C7	1.3072 (16)	C4—C5	1.389 (2)
N3—C9	1.2972 (17)	C4—H4A	0.9300
N3—N4	1.4222 (14)	C5—C6	1.387 (2)
N4—C11	1.3979 (16)	C5—H5A	0.9300
N4—C8	1.3988 (16)	C7—C9	1.4366 (17)
N5—C11	1.3185 (18)	C7—C8	1.4572 (17)
N5—H5B	0.8602	C9—C10	1.4880 (19)
N5—H5C	0.8600	C10—H10A	0.9600
C1—C2	1.383 (2)	C10—H10B	0.9600
C1—C6	1.3863 (19)	C10—H10C	0.9600
			
N2—N1—C6	119.64 (11)	C6—C5—H5A	120.7
N2—N1—H1B	120.2	C4—C5—H5A	120.7
C6—N1—H1B	120.2	C1—C6—C5	120.73 (13)
C7—N2—N1	119.04 (11)	C1—C6—N1	118.13 (12)
C9—N3—N4	107.04 (10)	C5—C6—N1	121.13 (12)
C11—N4—C8	130.31 (11)	N2—C7—C9	124.65 (12)
C11—N4—N3	117.83 (10)	N2—C7—C8	128.73 (12)
C8—N4—N3	111.70 (10)	C9—C7—C8	106.61 (10)
C11—N5—H5B	120.0	O1—C8—N4	129.69 (12)
C11—N5—H5C	120.0	O1—C8—C7	127.07 (12)
H5B—N5—H5C	120.0	N4—C8—C7	103.20 (10)
C2—C1—C6	119.56 (14)	N3—C9—C7	111.42 (11)
C2—C1—H1A	120.2	N3—C9—C10	122.83 (12)
C6—C1—H1A	120.2	C7—C9—C10	125.75 (12)
C3—C2—C1	120.38 (15)	C9—C10—H10A	109.5
C3—C2—H2A	119.8	C9—C10—H10B	109.5
C1—C2—H2A	119.8	H10A—C10—H10B	109.5
C2—C3—C4	119.73 (14)	C9—C10—H10C	109.5
C2—C3—H3A	120.1	H10A—C10—H10C	109.5
C4—C3—H3A	120.1	H10B—C10—H10C	109.5
C3—C4—C5	120.96 (16)	N5—C11—N4	113.45 (11)
C3—C4—H4A	119.5	N5—C11—S1	124.20 (10)
C5—C4—H4A	119.5	N4—C11—S1	122.34 (10)
C6—C5—C4	118.63 (15)		
			
C6—N1—N2—C7	179.04 (11)	C11—N4—C8—C7	173.49 (13)
C9—N3—N4—C11	−174.52 (12)	N3—N4—C8—C7	−1.73 (14)
C9—N3—N4—C8	1.36 (15)	N2—C7—C8—O1	4.4 (2)
C6—C1—C2—C3	−0.1 (2)	C9—C7—C8—O1	−176.61 (13)
C1—C2—C3—C4	−0.8 (3)	N2—C7—C8—N4	−177.54 (13)
C2—C3—C4—C5	1.2 (3)	C9—C7—C8—N4	1.46 (13)
C3—C4—C5—C6	−0.7 (2)	N4—N3—C9—C7	−0.33 (15)
C2—C1—C6—C5	0.6 (2)	N4—N3—C9—C10	179.58 (13)
C2—C1—C6—N1	−179.84 (13)	N2—C7—C9—N3	178.31 (12)
C4—C5—C6—C1	−0.2 (2)	C8—C7—C9—N3	−0.73 (15)
C4—C5—C6—N1	−179.78 (13)	N2—C7—C9—C10	−1.6 (2)
N2—N1—C6—C1	175.36 (12)	C8—C7—C9—C10	179.36 (14)
N2—N1—C6—C5	−5.05 (19)	C8—N4—C11—N5	174.38 (13)
N1—N2—C7—C9	−178.74 (12)	N3—N4—C11—N5	−10.64 (18)
N1—N2—C7—C8	0.1 (2)	C8—N4—C11—S1	−6.4 (2)
C11—N4—C8—O1	−8.5 (2)	N3—N4—C11—S1	168.56 (10)
N3—N4—C8—O1	176.27 (13)		

### Hydrogen-bond geometry (Å, °)

**Table:**

*D*—H···*A*	*D*—H	H···*A*	*D*···*A*	*D*—H···*A*
N1—H1B···O1	0.86	2.16	2.8147 (16)	132
N5—H5C···O1^i^	0.86	2.03	2.8806 (15)	172
C1—H1A···S1^ii^	0.93	2.80	3.6838 (16)	159

Symmetry codes: (i) *x*, −*y*+1/2, *z*−1/2; (ii) *x*, −*y*+1/2, *z*+1/2.
